# Efficacy of Radio Electric Asymmetric Conveyor (REAC) Anti-inflammatory, Circulatory, and Metabolic Optimization Treatments for Managing Chronic Pain, Edema, and Lipodystrophy in Post-menopausal Women: A Case Series

**DOI:** 10.7759/cureus.72653

**Published:** 2024-10-29

**Authors:** Ludmilla Higino Rocha, Alessandra Covallero Renck, Vania Fontani, Salvatore Rinaldi

**Affiliations:** 1 Anesthesiology, International Scientific Society of Neuro Psycho Physical Optimization With Radio Electric Asymmetric Conveyor (REAC) Technology, São Paulo, BRA; 2 Diabetes and Endocrinology, International Scientific Society of Neuro Psycho Physical Optimization With Radio Electric Asymmetric Conveyor (REAC) Technology, São Paulo, BRA; 3 Research Department, Rinaldi Fontani Foundation, Florence, ITA; 4 Department of Regenerative Medicine, Rinaldi Fontani Institute, Florence, ITA

**Keywords:** biomodulation, chronic inflammation, chronic pain, edema, lipodystrophy, metabolic dysfunctions, post-menopausal symptoms, reac biomodulation

## Abstract

Chronic pain, edema, and lipodystrophy are common issues in post-menopausal women, often linked to hormonal imbalances, metabolic dysfunctions, and chronic inflammation. Traditional treatments, such as hormone replacement therapy (HRT) and aesthetic procedures, frequently provide limited and temporary relief. This case series evaluates the effectiveness of radio electric asymmetric conveyor (REAC) technology specifically, the anti-cellulite treatment (ACT), circulatory optimization (CO), and metabolic optimization (MO) protocols in four post-menopausal women presenting with these conditions.

Each patient underwent 18 sessions of ACT, CO, and MO protocols using the REAC Bio-Enhancer Neuro-Enhancer (BENE) 110 medical device (ASMED Srl, Scandicci Florence, Italy), with asymmetric conveyor probes (ACPs) symmetrically positioned on the quadriceps. The ACT protocol targeted skin and subcutaneous tissue biomechanical properties, the CO protocol focused on enhancing circulation and reducing edema, and the MO protocol aimed to restore metabolic balance.

The results demonstrated significant improvements across all patients, with reductions in pain (9/10 to 3/10), edema, and lipodystrophy, as well as enhanced mood and overall well-being. Objective measures, such as reductions in thigh circumference and improvements in inflammatory markers, further supported the systemic benefits of REAC ACT, CO, and MO treatments. These findings suggest that REAC protocols offer a comprehensive and effective non-invasive treatment for chronic inflammatory and metabolic conditions in post-menopausal women.

## Introduction

Chronic pain, edema, and lipodystrophy are frequent and debilitating issues affecting post-menopausal women [1]. These symptoms often stem from the complex interplay between hormonal imbalances, metabolic disturbances, and chronic inflammation, leading to significant impairments in daily functioning, emotional well-being, and overall quality of life [2]. As estrogen levels decline, there is an increased risk of developing conditions such as metabolic syndrome [3], cardiovascular disease [4], and persistent inflammation [5]. Unfortunately, conventional treatments, including hormone replacement therapy [6], physical rehabilitation [7], and cosmetic procedures [8], often fail to provide long-term relief or adequately address the multifaceted nature of these conditions.

Due to these limitations, there is a growing demand for novel therapeutic interventions that offer both localized and systemic benefits. Radio electric asymmetric conveyor (REAC) technology, a promising development in neuromodulation [9-11] and biomodulation [12-15], has emerged as a potential solution. The REAC protocols, particularly anti-cellulite treatment (ACT), circulatory optimization (CO), and metabolic optimization (MO), provide a non-invasive approach to addressing the symptomatology of chronic pain, edema, and lipodystrophy [16,17]. These protocols not only target superficial aesthetic issues but also work to improve circulatory and metabolic functions, offering a comprehensive solution for conditions driven by chronic inflammation and hormonal imbalances.

This case series presents the results of applying REAC ACT, CO, and MO protocols in four post-menopausal women.

Methods

This case series involved four post-menopausal women, aged 45, 52, 58, and 59, who presented with chronic lower limb pain, persistent edema, and lipodystrophy. These conditions significantly impaired their quality of life for several years. The care was provided by specialized medical staff trained in REAC technology to ensure precise protocol application. The primary objective of this study was to assess the effectiveness of three specific REAC protocols: ACT, CO, and MO, in improving both clinical and symptomatic conditions in these patients [16,17].

The REAC protocols used in this study are designed to modulate cellular bioelectrical activity [18] by delivering weak radio electric fields asymmetrically conveyed. These protocols target the bioelectrical dysfunctions associated with inflammation, circulation, and metabolism. The ACT protocol aimed to reduce localized and systemic inflammation, which often exacerbates chronic pain, edema, and lipodystrophy, leading to improvements in skin elasticity and subcutaneous tissue properties. The CO protocol focused on enhancing blood circulation and optimizing tissue perfusion, which helps to alleviate edema and discomfort in the lower limbs. By improving microcirculation, this protocol also promotes oxygen delivery and reduces fluid retention. Finally, the MO protocol, administered after the CO protocol, was designed to restore metabolic balance, addressing metabolic dysregulation associated with chronic inflammation and circulatory dysfunction. This protocol helps improve fat distribution and overall metabolic health.

Each patient underwent 18 treatment sessions, with a minimum interval of one hour between sessions and a maximum of four sessions per day. The total treatment duration ranged from four to five weeks, depending on the individual case. The treatments were delivered using the REAC Bio-Enhancer Neuro-Enhancer (BENE) 110 medical device (ASMED Srl, Scandicci Florence, Italy), which is not a mechanical energy delivery device but instead asymmetrically conveys radio electric fields. The asymmetric conveyor probes (ACPs) were placed symmetrically on the quadriceps to ensure consistent therapeutic delivery. Each session lasted 15 minutes, and the treatments were administered sequentially, starting with the ACT protocol, followed by the CO protocol, and concluding with the MO protocol.

No additional treatments such as exercise or dietary modifications were introduced during this study, as the focus was solely on evaluating the effects of the REAC protocols.

Clinical evaluations were conducted at baseline and post-treatment to assess changes in symptoms such as pain, edema, and body composition, with particular attention to lipodystrophy. The ACT protocol targeted the improvement of the skin and subcutaneous tissues' biomechanical properties by reducing local inflammation and enhancing skin elasticity. The CO protocol aimed to improve blood circulation, tissue perfusion, and the reduction of edema, thereby addressing the discomfort and swelling in the lower limbs. The MO protocol focused on restoring metabolic balance, particularly in cases where chronic inflammation and circulatory dysfunction contributed to metabolic dysregulation. The MO protocol progressively improved fat distribution and overall metabolic health.

## Case presentation

The first patient, a 45-year-old woman, had been experiencing long-standing chronic pain, significant edema, and pronounced lipodystrophy in her lower limbs, all of which were exacerbated by premature menopause, which occurred at the unusually early age of 18. Her condition was further complicated by a chikungunya virus infection in 2023, leading to a worsening of her chronic pain, increased swelling in her legs, and a more pronounced appearance of lipodystrophy. Despite undergoing multiple aesthetic surgeries over the years, including rhinoplasty, mammoplasty, and liposuction, she found little relief from her symptoms. These procedures addressed some of the physical deformities caused by lipodystrophy, but they failed to provide the patient with long-term improvements in her overall physical health and functionality, leaving her with persistent pain and swelling. Although the patient's condition may resemble lipedema, the correct diagnosis in this case is lipodystrophy, as it involves abnormal fat distribution due to metabolic dysfunction, rather than the specific, symmetrical fat accumulation characteristic of lipedema.

Upon initiation of the REAC treatment protocols, the patient began a series of 18 sessions comprising the ACT, CO, and MO treatments, each designed to address different aspects of her condition. The ACT protocol focused on improving skin elasticity and the biomechanical properties of subcutaneous tissues, particularly in areas affected by lipodystrophy. The CO protocol targeted the underlying circulatory deficiencies, aiming to enhance tissue perfusion, alleviate edema, and reduce the frequency of nighttime cramps that had been significantly impairing her mobility. Finally, the MO protocol was designed to restore metabolic balance by targeting the underlying metabolic dysfunctions exacerbated by chronic inflammation, thereby helping to reduce the systemic effects of such inflammation.

**Figure 1:**
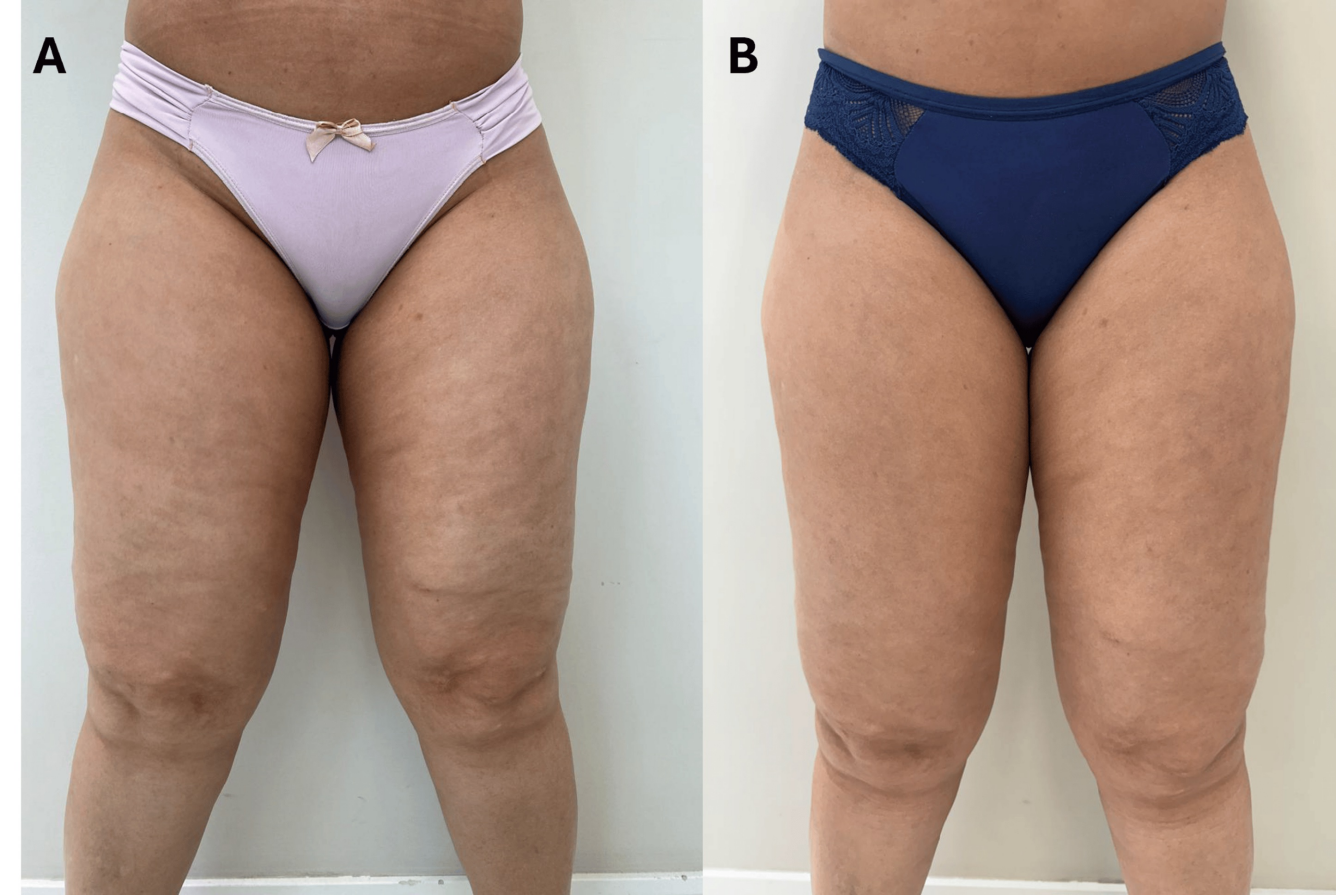
Pre- and post-treatment impact of REAC ACT, CO, and MO protocols on chronic pain, edema, and lipodystrophy. (A) (Pre-treatment): Shows significant edema and lipodystrophy, with pain rated at 9/10. (B) (Post-treatment): Demonstrates reduced edema, improved skin firmness, and a pain reduction to 3/10 after 18 REAC sessions. REAC: radio electric asymmetric conveyor; ACT: anti-cellulite treatment; CO: circulatory optimization; MO: metabolic optimization

Following treatment, the patient reported a significant reduction in her pain levels, with intensity decreasing from 9/10 to 3/10. She also experienced noticeable improvements in the firmness and appearance of her legs, as subjectively reported by the patient and observed by the clinical team. However, no objective scales or standardized measurements were used to quantify these changes, which limits the generalizability of these findings. The alleviation of edema led to improved mobility, and the reduction of nighttime cramps allowed her to regain a more active lifestyle. However, no specific measures such as bioimpedance or limb circumference were taken to assess the reduction in edema, as the primary goal of the study was to focus on overall well-being and functional improvements in post-menopausal women, rather than on aesthetic outcomes. Beyond the physical benefits, the patient’s mood, energy levels, and overall well-being improved markedly, with objective measures further supporting these outcomes. Clinical indicators of inflammation, such as the reduction of pain, swelling, and edema, provided concrete evidence of the systemic benefits of the REAC treatments. These outcomes illustrate how these protocols effectively addressed both the superficial and deep-seated aspects of her condition. However, no blood-based inflammatory markers were measured in this case (Figure 1A-1B).

The second patient, aged 52, had been suffering from chronic lower limb pain and lipodystrophy for over two decades. Her symptoms had steadily worsened over time, compounded by emotional distress and recurrent episodes of depression, which she had attempted to manage with various medications including anxiolytics, antidepressants, mood stabilizers, atypical antipsychotics, nonsteroidal anti-inflammatory drugs (NSAIDs), and analgesics as well as countless types of aesthetic approaches. Notably, she had also tried weight loss drugs, including Ozempic, without significant long-term success. The persistence of her condition, despite attempts to control it through both pharmacological and non-pharmacological means, had significantly impacted her quality of life.

Following the initiation of the 18-session REAC ACT, CO, and MO protocols, the patient began to experience significant improvements. The ACT protocol helped address the lipodystrophy and poor skin texture that had long been a source of emotional distress. The CO protocol, by enhancing circulation, contributed to a reduction in the sensation of heaviness in her legs and improved her overall mobility. Notably, this protocol also appeared to accelerate her recovery from a rib injury. Her surgeon commented that the speed of healing was unusually rapid, suggesting that the improvements in tissue perfusion achieved through the REAC protocols may have contributed to enhanced recovery and tissue repair.

**Figure 2:**
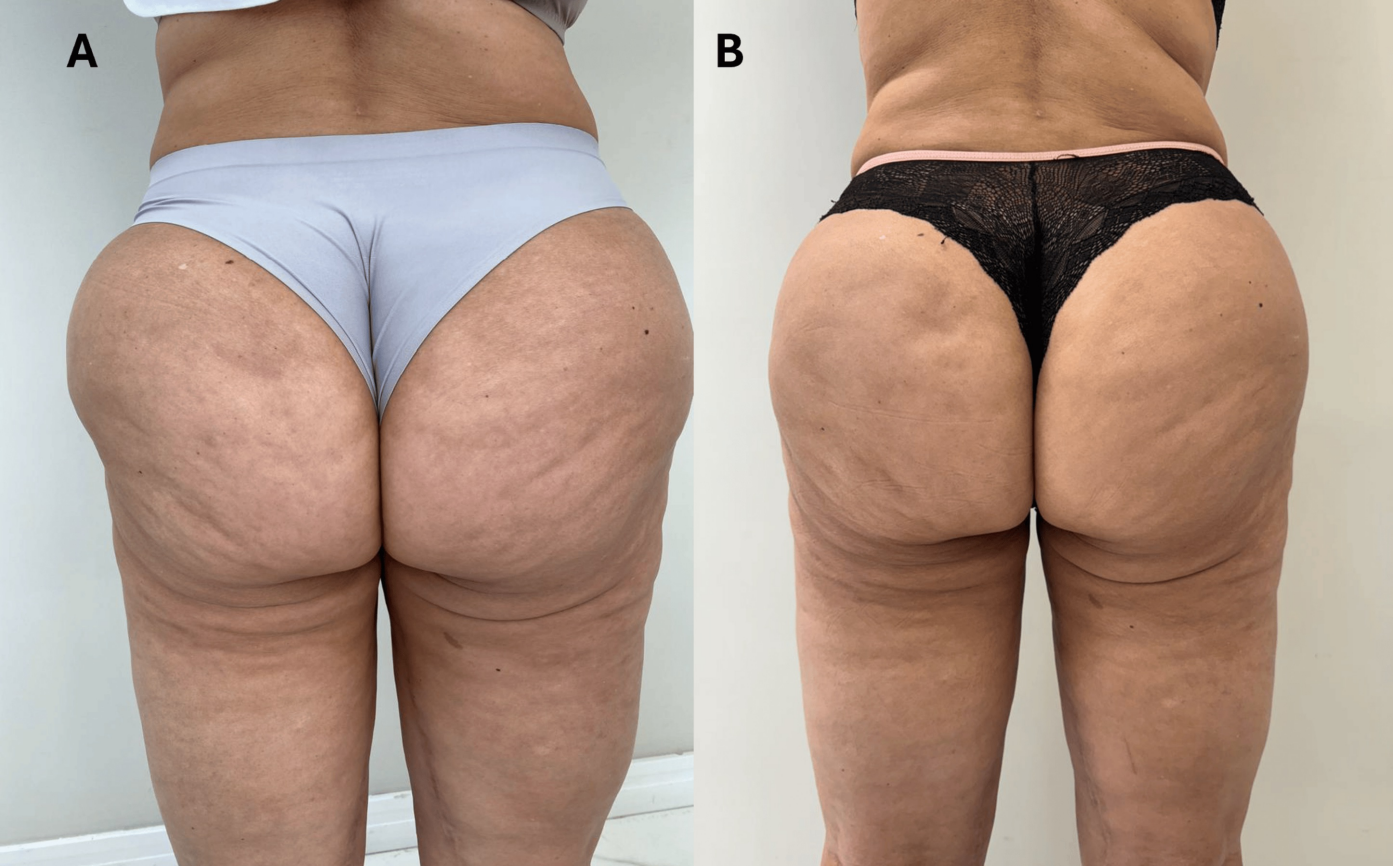
Pre- and post-treatment effects of REAC ACT, CO, and MO protocols in pain and limb heaviness. (A) (Pre-treatment): Severe pain (10/10) and leg heaviness due to edema and lipodystrophy. (B) (Post-treatment): Pain reduced to 1/10 with visible improvements in limb appearance and reductions in circumferences. REAC: radio electric asymmetric conveyor; ACT: anti-cellulite treatment; CO: circulatory optimization; MO: metabolic optimization

**Figure 3:**
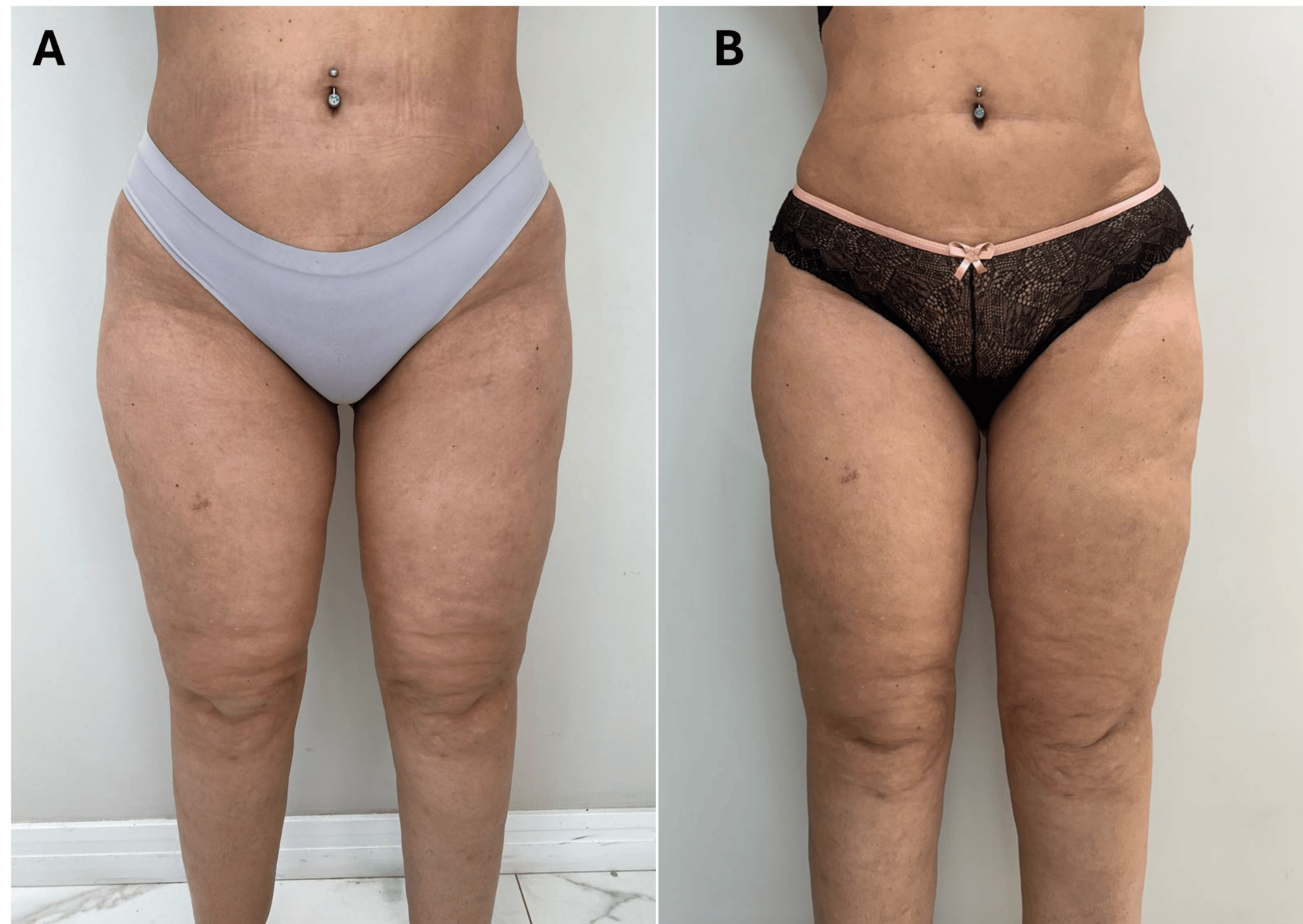
Pre- and post-treatment effects of REAC ACT, CO, and MO protocols in pain and limb heaviness. (A) (Pre-treatment): Visible lipodystrophy, poor skin texture, and emotional distress. (B) (Post-treatment): Improved skin texture and emotional stability, with reduced body measurements. REAC: radio electric asymmetric conveyor; ACT: anti-cellulite treatment; CO: circulatory optimization; MO: metabolic optimization

The patient’s pain levels decreased from 10/10 to 1/10, and she noticed improvements in her sleep quality, which in turn enhanced her emotional stability and overall mood. The MO protocol further reduced water retention, leading to a slimmer and more contoured appearance, as demonstrated by the reduction in both abdominal and thigh circumferences. These objective measurements were complemented by a boost in self-esteem, as the patient no longer relied on medication to manage her emotional and physical health. The REAC treatments provided her with lasting relief, significantly improving her daily functioning and sense of well-being (Figures 2A-2B, 3A-3B).

The third patient, a 59-year-old woman, presented with a complex medical history that included scoliosis, peripheral insulin resistance, and phantom pain following maxillofacial surgery. These pre-existing conditions had led to chronic hip pain, lower limb edema, and severe mobility limitations. The patient had struggled with pain management for years, relying on various treatments that had provided minimal relief. The combination of chronic hip pain and persistent edema had made it increasingly difficult for her to perform even the most basic tasks, such as dressing or standing for extended periods. Her overall physical health was in decline, and she expressed frustration over her limited mobility and diminished quality of life.

After completing 18 sessions of the REAC ACT, CO, and MO protocols, the patient reported substantial improvements in both her physical condition and emotional well-being. The ACT protocol resulted in a rapid reduction in hip pain, enabling her to regain muscle strength and perform activities she had previously been unable to, such as dressing while standing. The CO protocol further alleviated the lower limb edema that had long impaired her mobility, and it also allowed her to engage in more physically demanding activities without the discomfort she had previously experienced.

**Figure 4:**
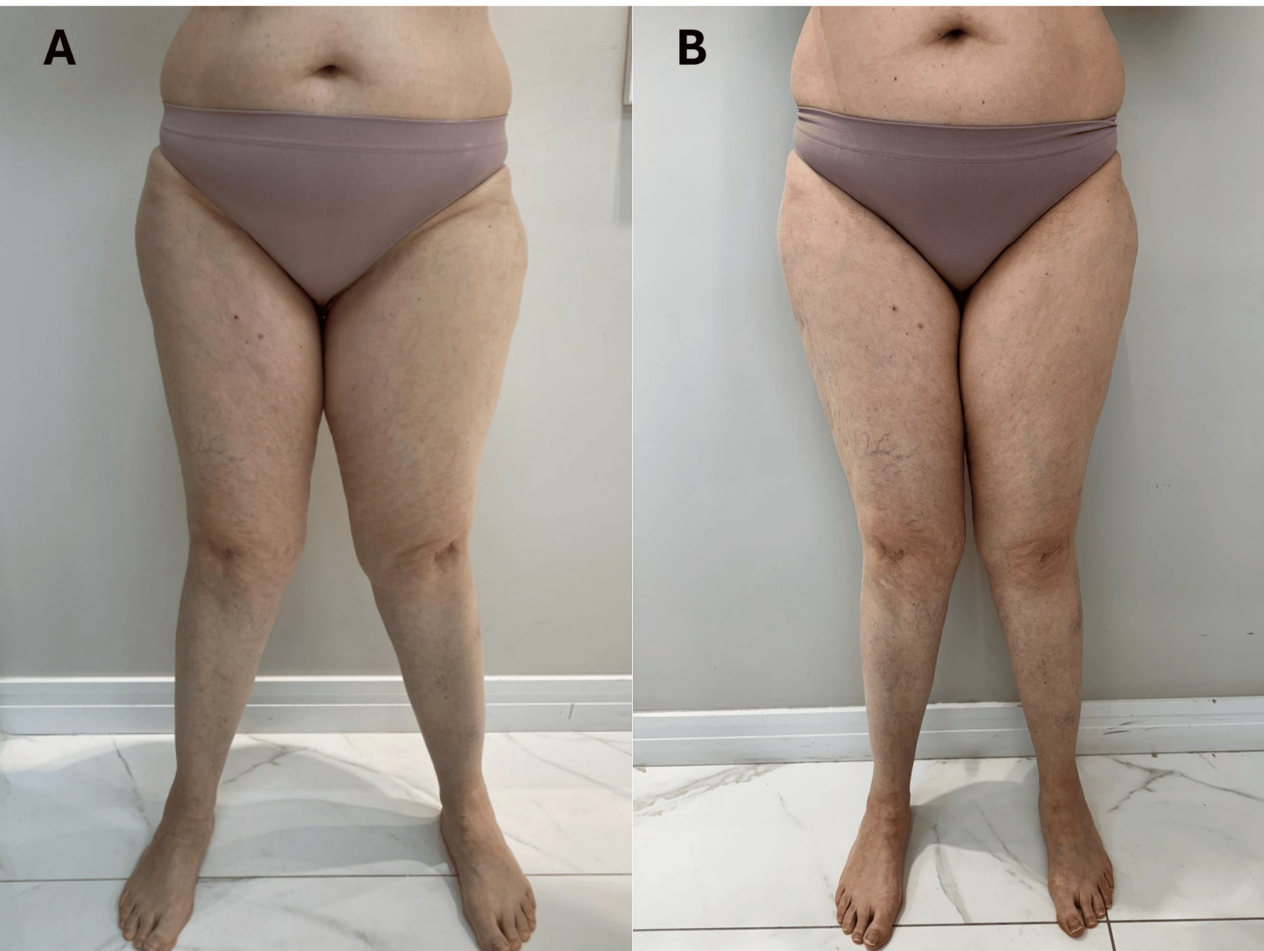
Impact of REAC protocols on hip pain and mobility. (A) (Pre-treatment): Shows chronic pain (10/10), lower limb edema, and mobility limitations. (B) (Post-treatment): Reduced pain (3/10) and improved limb symmetry and mobility after treatment. REAC: radio electric asymmetric conveyor

The MO protocol helped improve her metabolic profile, contributing to a significant reduction in lower limb swelling and an increase in skin elasticity. Additionally, her endurance improved, allowing her to engage more freely in daily tasks. Pain severity dropped from 10/10 to 3/10, and objective measurements revealed a reduction in thigh asymmetry, providing further evidence of the therapeutic efficacy of the REAC protocols. Moreover, the patient reported improved sleep quality and a reduction in anxiety, underscoring the comprehensive benefits of the treatments. The positive outcomes in this complex case illustrate the ability of REAC protocols to provide significant relief even in patients with multifactorial health challenges (Figure 4A-4B).

The fourth patient, a 58-year-old post-menopausal woman, presented with a multifaceted clinical history that included metabolic syndrome, chronic fatigue, generalized pain, and emotional disturbances. Since entering menopause at age 51, she had gained 10 kilograms, leading to pre-diabetes, dyslipidemia, and non-alcoholic fatty liver disease (NAFLD). Additionally, she suffered from right knee pain due to chondromalacia, requiring daily NSAID use. The patient also reported anxiety and depression, managed with paroxetine, alongside her use of metformin extended-release (XR) (500 mg/day) to control her metabolic condition. Her main concerns were chronic fatigue, abdominal fat accumulation, generalized pain, and poor sleep quality, which significantly impaired her quality of life.

**Figure 5:**
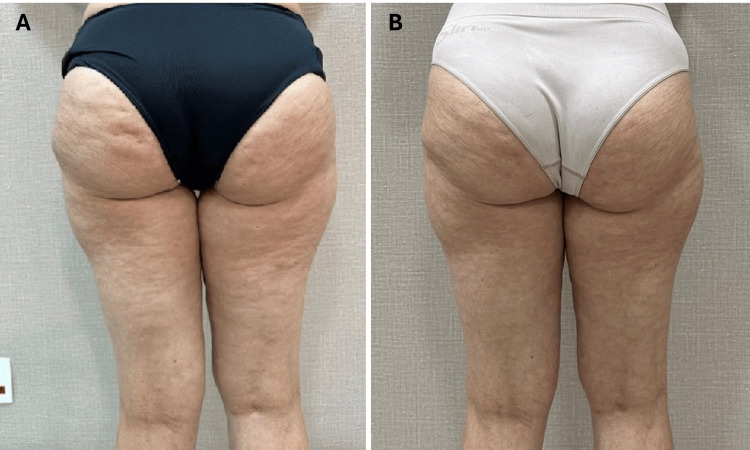
Before and after REAC ACT, CO, and MO treatments in a 58-year-old post-menopausal woman. (A) Before treatment, with visible signs of lipodystrophy and uneven skin texture. (B) After completing the full treatment cycle, showing improved skin firmness, reduced lipodystrophy, and a more contoured appearance. REAC: radio electric asymmetric conveyor; ACT: anti-cellulite treatment; CO: circulatory optimization; MO: metabolic optimization

Over the course of treatment, the patient experienced substantial improvements. By the fourth session of CO, she reported enhanced physical agility, the resolution of dizziness, and improved thoracic relaxation. Importantly, her sleep quality improved significantly, leading to an overall better sense of well-being. By the end of the full treatment cycle, the patient observed a 4 cm reduction in abdominal circumference and increased muscle mass (Figure 5A-5B).

These physical improvements were accompanied by a decrease in her reliance on both NSAIDs for pain management and paroxetine for emotional regulation, marking a crucial shift in her health management.

This case stands out due to the patient’s marked reduction in medication dependency, which reflects the broader systemic benefits of the REAC treatments. The reduction of inflammation, improvement in metabolic regulation, and emotional stabilization allowed for substantial improvements in both her physical and emotional well-being.

## Discussion

This case series highlights the effectiveness of REAC technology, particularly the ACT, CO, and MO protocols, in managing complex post-menopausal symptoms, including chronic pain, edema, lipodystrophy, and metabolic dysfunction [16,17]. These protocols provide a non-invasive, comprehensive therapeutic approach, addressing both localized symptoms and broader systemic dysfunctions [16,17]. A significant observation from this series is the reduction in medication dependency, with patients showing less reliance on NSAIDs and antidepressants, which emphasizes the potential of REAC treatments as an alternative to pharmacological interventions. Additionally, the rapid recovery of tissue injury, as seen in some cases, suggests REAC protocols may have reparative and regenerative properties beyond symptom management. Compared to conventional therapies like hormone replacement therapy (HRT) [6,19,20], which carry risks, REAC offers a safer, non-invasive solution that addresses both aesthetic and systemic issues like inflammation and metabolic imbalance. Future research should explore long-term sustainability of these benefits, as well as the broader potential of REAC technology in managing other chronic conditions and its application in reparative and regenerative medicine.

## Conclusions

These results are based primarily on subjective reports. Future studies should incorporate objective markers, such as serum inflammatory markers, lipodystrophy-related measurements, and quality-of-life assessments to provide more robust support for these outcomes. In conclusion, this case series provides valuable new insights into the therapeutic potential of REAC ACT, CO, and MO protocols. The results underscore the versatility and effectiveness of these treatments in addressing both the physical and emotional aspects of post-menopausal conditions. By offering a comprehensive, non-invasive solution, the REAC technology represents a significant advancement in the field of reparative medicine. Future research with larger patient populations and longer follow-up periods will further validate these findings and explore additional therapeutic applications.
